# Machine learning: An effective technical method for future use in assessing the effectiveness of phosphorus-dissolving microbial agroremediation

**DOI:** 10.3389/fbioe.2023.1189166

**Published:** 2023-03-31

**Authors:** Juai Wu, Fangzhou Zhao

**Affiliations:** ^1^ College of Automation & College of Artificial Intelligence, Nanjing University of Posts and Telecommunications, Nanjing, China; ^2^ School of Environmental and Biological Engineering, Nanjing University of Science and Technology, Nanjing, China

**Keywords:** machine learning, microbial remediation, agricultural pollution, assessment and prediction, crop yield

## Abstract

The issue of agricultural pollution has become one of the most important environmental concerns worldwide because of its relevance to human survival and health. Microbial remediation is an effective method for treating heavy metal pollution in agriculture, but the evaluation of its effectiveness has been a difficult issue. Machine learning (ML), a widely used data processing technique, can improve the accuracy of assessments and predictions by analyzing and processing large amounts of data. In microbial remediation, ML can help identify the types of microbes, mechanisms of action and adapted environments, predict the effectiveness of microbial remediation and potential problems, and assess the ecological benefits and crop growth after remediation. In addition, ML can help optimize monitoring programs, improve the accuracy and effectiveness of heavy metal pollution monitoring, and provide a scientific basis for the development of treatment measures. Therefore, ML has important application prospects in assessing the effectiveness of microbial remediation of heavy metal pollution in agriculture and is expected to be an effective pollution management technology.

## 1 Introduction

The safety of agricultural land as the basis for human survival and development is of increasing concern (Roy et al., 2022). In recent years, the levels of heavy metals in agricultural land have been increasing due to industrial activities, fertilizer application, coal burning, and other human activities (Xu et al., 2014). Heavy metals are characterized by non-biodegradability, high toxicity, and ease of accumulation, and are the most common type of pollutants in the agricultural environment, seriously affecting ecosystem function, food security, and human health (Sharma et al., 2022). In addition, higher levels of heavy metals may lead to changes in soil structure and nutrient loss, which in turn may affect crop quality and yield (Fei et al., 2022). Therefore, it is necessary to analyze the effects of heavy metal pollution on agricultural land and to assess the level of heavy metal pollution in agricultural land.

## 2 Phosphate solubilizing microorganisms as a green technology for improving crop growth and remediating heavy metal contamination

Microorganisms play an important role in the growth of crops in agricultural fields, and can enhance crop growth by improving the soil environment, promoting nutrient cycling, and improving plant immunity in a variety of ways. Phosphorus solubilizing microorganisms (PSM) are a group of microorganisms that can convert soil organic phosphorus compounds or insoluble inorganic phosphorus compounds into plant-available phosphorus elements. As one of the first beneficial functional microorganisms discovered in agricultural fields, the main functions of PSM include 1) increasing the effective soil phosphorus content through the release of organic acids, biological enzymes, and a series of other secretions to promote plant nutrient uptake and growth and development (Alori et al., 2017). 2) Forming a symbiotic relationship with plant roots, using inter-root substances secreted by plants for subsistence, as well as secreting hormones and enzymes (indoleacetic acid, 1-Aminocyclopropane-1-carboxylate deaminase, *etc.*) to help plants absorb nutrients (Rawat et al., 2020). 3) Produce plant antibodies (e.g., cell wall lysis enzymes, antibiotics, *etc.*) to suppress plant diseases, activate the plant’s immune system, and enhance plant resistance to diseases (Zaidi et al., 2016). 4) Decomposing organic matter and promoting soil aeration and water retention capacity, thus improving soil structure and nutrient retention (Tian et al., 2021). 5) Phosphate solubilizing microorganisms are able to adsorb, accumulate and complex heavy metal ions as well as toxicity reduction through cellular self and secretions (extracellular polymers, glutathione, *etc.*) (Chen et al., 2023b). 6) The use of phosphate solubilizing microorganisms can also reduce the use of chemical fertilizers and pesticides, reducing the negative impact on the environment (Ahemad, 2015). In addition, the good affinity of PSM allows it to be used together with other materials to improve soil properties and remediate environmental pollution (Chen et al., 2019; Feng et al., 2022; Lai et al., 2022; Chen et al., 2023a).

## 3 Artificial intelligence—machine learning has been initially applied in agricultural environments at this stage

Soil contamination assessment in agriculture is a prerequisite to ensure that crops can be grown properly and safely consumed, and the classical research paradigms include three types: experiment, theory, and computational science. Traditional methods for land contamination assessment include the single factor index, single factor pollution index, potential ecological risk index, and contamination load index (El Azhari et al., 2017; Lu et al., 2021). These methods show some limitations in practical applications due to the lack of adaptability and accuracy of the built mathematical models. The era of big data has given rise to the data-driven paradigm based on big data, which refers to data sets with complex structures, including those that do not have or have not yet mastered their causal relationships. The core of the data-driven approach lies in acquiring knowledge by analyzing large amounts of data. Common data analysis methods include classification, clustering, association (correlation, regression, *etc.*), discrimination, principal component analysis, statistical inference, *etc.* (Wu et al., 2020).

In recent years, data mining methods using artificial intelligence algorithms such as machine learning have become a hot research topic. Machine learning method is an advanced data analysis method, which is often applied to analyze the hidden information between input data and output results (Dobbelaere et al., 2021). An integrated model composed of multiple learning algorithms has the advantages of good predictive performance and high interpretability (Zhang et al., 2022). For example, random forest can directly process high-dimensional data without feature selection (Ali et al., 2021). Gradient boosting decision tree use a loss function with robustness to outliers (Yang et al., 2023). These models have been successfully applied to problems such as forecasting, engineering design, and material optimization, saving significant labor and time costs (Tian et al., 2022; Veloso et al., 2022). Currently, machine learning is widely used in agriculture. For example, Hamrani et al. successfully predicted greenhouse gas emissions in agricultural land using nine models separately, such as support vector machine, random forest, *etc.* Among them, long short-term memory network has the most accurate prediction for both CO_2_ (*R* ^2^ = 0.87, RMSE = 30.3) and N_2_O (*R* ^2^ = 0.86, RMSE = 0.19) (Hamrani et al., 2020). Saha et al. combined a machine learning model and multi-criteria decision-making models to assess agricultural land fertility and site suitability, it was found that agricultural land with higher organic carbon content and cation exchange capacity, and low bulk weight was more advantageous (Saha and Mondal, 2022). Some studies have also shown that clay content in the soil, soluble phosphorus, and soluble organic carbon are the main factors affecting phosphorus concentration in groundwater of agricultural land using support vector machine, random forest, and neural network (Yang et al., 2023). These successful examples show that it is feasible to use machine learning methods for prediction, assessment, and analysis of pollution problems in agricultural environments.

## 4 Building machine learning models to predict crop yield and safety after remediation of heavy metal contamination is one of the hot spots for future research

**FIGURE 1:**
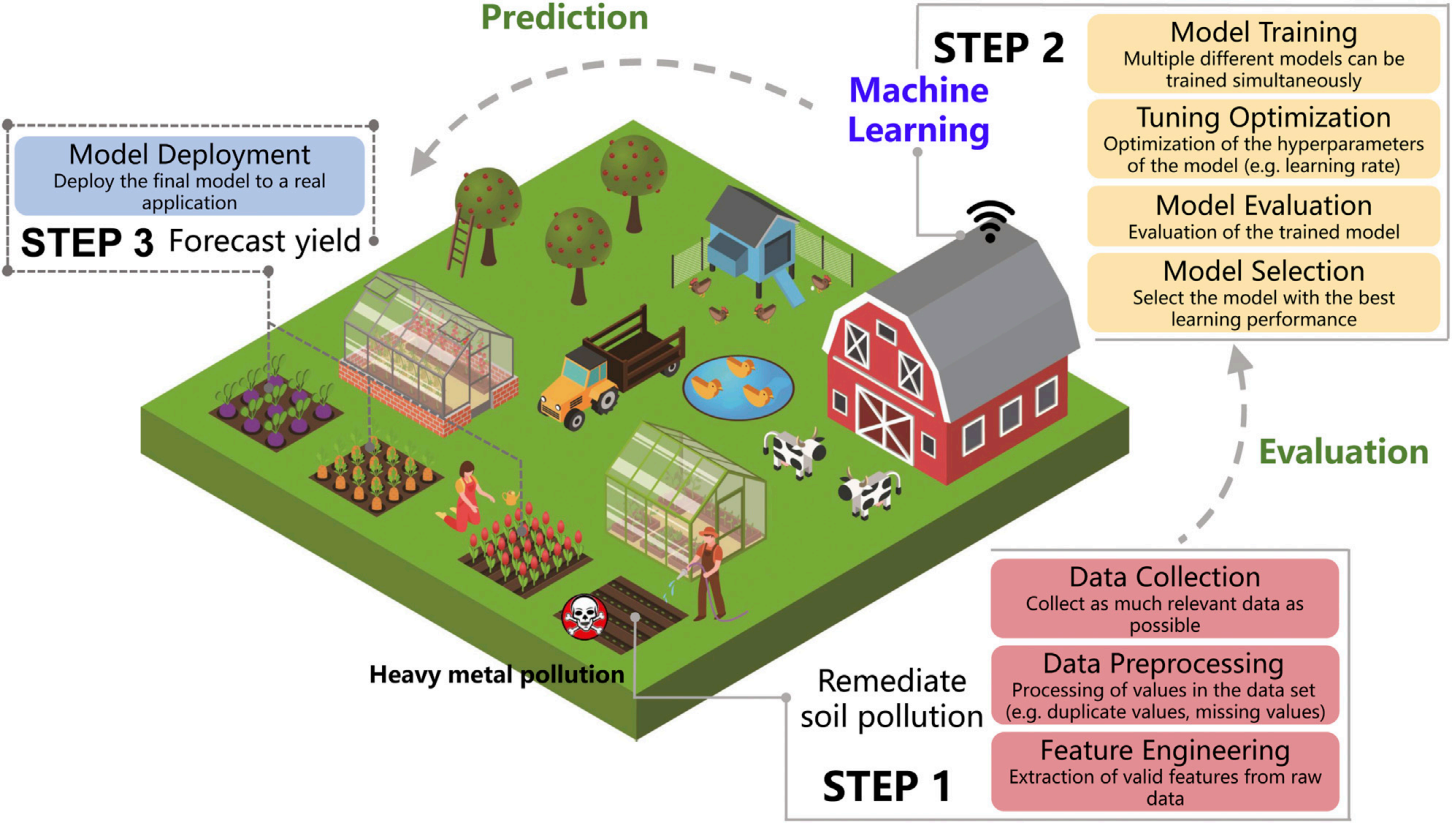
Application pattern diagram of machine learning model in heavy metal pollution remediation in agriculture.

In agricultural land, crop yield is influenced by several factors, including heavy metal contamination concentrations, nutrient elements in the soil, and microbial communities. These factors also have some mechanisms of action among each other. Currently, it is difficult to quantify the effects of these mechanisms of action on crop yield. Therefore, it has become a trend to use machine learning models to simulate and assess soil contamination levels (Wang et al., 2021; Liu et al., 2023). Based on the powerful data analysis methods of machine learning, and integrating the data-driven method (statistical analysis) with the model-driven method (causal analysis), it is possible to link heavy metal contamination with crop yield. It would be an effective technical tool to explore the effect mechanism of remediated heavy metal contamination on nutrient elements, microorganisms and crop yield. The analysis diagram is shown in Figure 1.

Based on the excellent predictive performance of machine learning models, it is feasible to build a model to predict crop yield. Typical machine learning model structures include supervised learning represented by regression analysis and statistical classification, unsupervised learning represented by generating adversarial network (GAN) and clustering, and reinforcement learning (LeCun et al., 2015; Castaldo et al., 2016; Wang et al., 2020). Currently, deep learning represented by neural network is rapidly developing, which would be more efficient to integrate with supervised learning, unsupervised learning, and reinforcement learning to classify data and identify features. Due to the differences in the datasets, the prediction performance of each model behaves differently. During the model construction process, multiple models are usually selected for simultaneous parameter optimization and prediction, and the model with the best prediction performance is finally selected. Besides, a sufficient amount of data is also one of the important factors to support the success of model prediction. For example, Jhajharia et al. integrated 3,664 sets of yield data of various crops with soil type and rainfall of agricultural land in the Rajasthan region, and the results confirmed that crop yield could be successfully predicted using random forest model (*R* ^2^ = 0.963, RMSE = 0.035) (Jhajharia et al., 2023). Iniyan et al. counted data on precipitation, humidity, temperature, area, soil type, crop type, season, and yield for the last 18 years in the Maharashtra region. After training eight machine learning models such as linear regression, ridge regression, and gradient boosting, it was found that the long short-term memory network showed the best prediction performance with 86.3% accuracy (Iniyan et al., 2023). Panigrahi et al. successfully used linear regression, decision tree regression, and other models to predict the yield of three different crops of corn, peanuts, and Bengal beans based on the monthly minimum and maximum temperature and annual rainfall in the Telangana (Panigrahi et al., 2023). Therefore, it is feasible to predict the yield of various crops using machine learning models, but the availability of sufficient data support from research institutions or government may be the key to it.

Machine learning models can not only predict crop yields, but also analyze the effect of PSM on remediation of heavy metal contamination or post-remediation soil properties on crop yields through interpretable algorithms such as feature importance, partial dependence, individual conditional expectation, and Shapley additive explanation. For example, by analyzing the partial dependence of a certain heavy metal ion, the concentration threshold for that heavy metal to affect crop growth is determined. Alternatively, using the characteristic importance algorithm, it is possible to obtain the importance weights for the effects of various types of heavy metals on crop yield. Such weighting factors, which can be introduced into the formula for calculating the risk level of heavy metal contamination, make the assessment results more informative. In addition, the joint application of machine learning and high-resolution aerial imaging technology has been shown to be feasible. For example, feature information is obtained based on high-resolution aerial imaging technology, and then machine learning models are used to predict the heavy metal concentrations at unknown points to finally assess the soil contamination risk levels. The accuracy of this combined application of techniques to assess contamination levels was verified to be significantly better than the traditional kriging interpolation and inverse distance weight interpolation methods (Jia et al., 2021). In conclusion, machine learning models can simultaneously combine soil quality and crop yield so that the two are interlinked, which means that the impact of soil quality on crop growth can be analyzed along with predicting crop yield, correcting adverse factors that affect yield and continuously improving agricultural land quality. Thus, machine learning can be used as an effective technical tool to predict and evaluate the impact of phosphorus dissolving microbially remediated soils on crop yields.

## 5 Discussion

As an efficient and fast data analysis and processing method, machine learning has often been applied to solve various agricultural environmental problems in recent years. Its powerful prediction and analysis capability saves a lot of labor cost and time cost for research, and the application of machine learning in agricultural survey technology will be more promising in the future with the advancement of technology and the increase of data. However, the application direction of the technology is currently more limited. In the future, artificial intelligence, machine learning, and computer vision can be used to identify the growth status of crops and prevent them from being infected with toxic pests to affect their yield. On the other hand, machine learning is essentially a data-driven method, there is currently less training data available to researchers. A large amount of open-source data is also necessary for training models in future research. In this context, data-driven method and model-driven method can complement each other, which includes: 1) introduce casual analysis measures to researches that usually relied entirely on statistical analysis to solve data dependency and improve the analysis applicability and accuracy; 2) introduce statistical analysis measures to researches that usually relied entirely on casual analysis, to improve the analysis efficiency.

In conclusion, machine learning can be used to achieve accurate analysis and prediction of agricultural data, improve the efficiency and quality of agricultural production, and promote the development and upgrading of the agricultural industry.

## Data Availability

The original contributions presented in the study are included in the article/Supplementary Material, further inquiries can be directed to the corresponding author.
